# Community Structure of Bacteria Associated With Drifting *Sargassum horneri*, the Causative Species of Golden Tide in the Yellow Sea

**DOI:** 10.3389/fmicb.2019.01192

**Published:** 2019-05-28

**Authors:** Xiangyuan Mei, Chunhui Wu, Jin Zhao, Tian Yan, Peng Jiang

**Affiliations:** ^1^CAS Key Laboratory of Experimental Marine Biology, Institute of Oceanology, Chinese Academy of Sciences, Qingdao, China; ^2^Laboratory for Marine Biology and Biotechnology, Qingdao National Laboratory for Marine Science and Technology, Qingdao, China; ^3^Center for Ocean Mega-Science, Chinese Academy of Sciences, Qingdao, China; ^4^University of Chinese Academy of Sciences, Beijing, China; ^5^CAS Key Laboratory of Marine Ecology and Environmental Sciences, Institute of Oceanology, Chinese Academy of Sciences, Qingdao, China; ^6^Laboratory for Marine Ecology and Environmental Science, Qingdao National Laboratory for Marine Science and Technology, Qingdao, China

**Keywords:** 16S rRNA gene, high-throughput sequencing, bacterial community, golden tide, *Sargassum horneri*, the Yellow Sea

## Abstract

Golden tides dominated by *Sargassum* spp. are occurring at an accelerated rate worldwide. In China, *Sargassum* has started to bloom in the Yellow Sea and led to tremendous economic losses, but the underlying biological causes and mechanisms are still unclear. Although algae-associated bacteria were suggested to play crucial roles in algal blooms, the profiles of bacteria associated with drifting *Sargassum* remain unexplored. In this study, the community structures and functions of *Sargassum*-associated bacteria were analyzed using the high-throughput sequencing data of the V5–V7 hypervariable region of the 16S rRNA gene. Molecular identification revealed that the golden tide analyzed in the Yellow Sea was dominated by a single species, *Sargassum horneri*. They were a healthy brown color nearshore but were yellow offshore with significantly decreased chlorophyll contents (*P* < 0.01), which indicates that yellow *S. horneri* was under physiological stress. The structural and functional analyses of bacterial communities indicated that the drifting *S. horneri* had an obvious selectivity on their associated bacteria against surrounding seawater. Although the bacterial communities phylogenetically differed between brown and yellow *S. horneri* (*P* < 0.01), their dominant functions were all nitrogen and iron transporters, which strongly indicates microbial contribution to blooming of the algal host. For the first time, potential epiphytic and endophytic bacteria associated with *Sargassum* were independently analyzed by a modified co-vortex method with silica sand. We showed that the composition of dominant endophytes, mainly *Bacillus* and *Propionibacterium*, was relatively consistent regardless of host status, whereas the epiphytic operational taxonomic units (OTUs) greatly varied in response to weakness of host status; however, dominant functions were consistent at elevated intensities, which might protect the host from stress related to nitrogen or iron deficiency. Thus, we propose that host physiological status at different intensities of functional demands, which were related to variable environmental conditions, may be a critical factor that influences the assembly of epiphytic bacterial communities. This study provided new insight into the structure and potential functions of associated bacteria with golden tide blooms.

## Introduction

Golden tides are harmful algal blooms that are occurring at an accelerated rate worldwide and are caused by rapid proliferation of drifting brown seaweed *Sargassum* (Smetacek and Zingone, 2013; Sissini et al., 2017). For several decades, drifting *Sargassum horneri* aggregations have been reported in the East China Sea, but its origin remains unknown (Komatsu et al., 2014b; Qi et al., 2017). However, at the end of 2016, a large-scale golden tide bloomed in the adjacent Yellow Sea for the first time and destroyed nearshore *Pyropia yezoensis* cultivation, which resulted in substantial economic losses of as much as 500 million CNY (approximately 73 million United States dollars) (Xing et al., 2017). It was suggested that golden tides along coastal China might undergo notable variation, and the mechanism of blooming needs to be clarified for future disaster management.

Although up to 17 local *Sargassum* species are distributed along the coast of the Yellow Sea and East China Sea, which are fixed to substrate (Lu and Tseng, 2004; Huang et al., 2017), *S. horneri* was identified as the only dominant species of drifting *Sargassum* in the Yellow Sea (Su L. et al., 2018). This fact indicated that, except environmental factors such as nutrient content or ocean current, the distinct biological causes of *S. horneri* were also worthy of special attention. To date, the floating mechanism of *S. horneri* has been preliminarily studied, and it was found that the vesicles provide the floating force (Komatsu et al., 2014b), and the degree of maturity affects the detachment of the thallus from substrate (Xu et al., 2016); however, the cause of golden tides is still unclear.

Algae–bacteria interactions have been widely reported. As a host, algae provide habitat, oxygen, and carbohydrates such as algal polysaccharides for associated bacteria. In turn, bacteria provide hormones, vitamins, minerals, and carbon dioxide to algae, thus playing important roles in algal morphogenesis, growth, immune defense, and even spore release and germination (Matsuo et al., 2003; Croft et al., 2005; Singh and Reddy, 2014; Cho et al., 2015; Kouzuma and Watanabe, 2015). In addition, the associated bacteria also greatly contribute to algal bloom initiation and maintenance by secreting growth-promoting substances (Park et al., 2016), participating in nitrogen metabolism through nitrogen fixation (Falcon et al., 2002), or dissimilatory nitrate reduction to ammonium (DNRA), which would benefit the blooming of algal host from nutrient competition (An and Gardner, 2002). Therefore, it was suggested that the analyzing on associated bacteria may provide new insights into the causes and dynamics of seaweed blooming (Fu et al., 2018).

However, almost all related studies were performed on blooming microalgae, such as diatoms, dinoflagellates, or cyanobacteria (Sison-Mangus et al., 2016; Berry et al., 2017; Parulekar et al., 2017), and only a few studies have examined blooming macroalgae. Additionally, only epiphytic bacterial communities were preliminarily analyzed by denaturing gradient gel electrophoresis (DGGE) in green tide seaweed (Liu et al., 2011). To clarify the community structure and functions of bacteria associated with drifting *Sargassum*, three issues needed to be addressed.

### Selectivity

Bacterial communities associated with attached macroalgae are obviously distinct from those in ambient seawater (Tujula et al., 2010; Burke et al., 2011b; Michelou et al., 2013) and also various among macroalgae species (Longford et al., 2007; Lachnit et al., 2009). This strong selectivity indicated specific functional requirements that seaweed imposed on associated bacteria, especially in relatively stable environments (Burke et al., 2011a; Zhang et al., 2018). However, drifting *Sargassum* experience rapid changes of both ambient conditions and physiological status (Komatsu et al., 2014a); therefore, whether the selectivity on associated bacteria still exists and varies under these circumstances still needs to be illustrated.

### Spatial Distribution

Preliminary tissue differentiation occurs in brown seaweed, and variation in bacterial communities among tissues has been noticed (Goecke et al., 2010). *Sargassum* possesses highly specialized structures, such as vesicles and blades with distinct adaptive functions for floating, but it was unknown whether the associated bacteria differed among tissues. In addition, almost all previous studies analyzed epiphytes rather than endophytes. To date, endophytic bacteria were mainly reported in red and green seaweed (Hollants et al., 2011b; Singh et al., 2011; Aires et al., 2015), and usually have a simple and stable composition (Hollants et al., 2011a). A comparative genomic analysis of an endophytic isolate from *Ulva prolifera* implied that endophytes might benefit hosts from nitrogen competition during blooming (Fu et al., 2018). Alternatively, similar research has not been carried out on brown seaweed, because of a lack of available methods for obtaining endophytic bacteria.

### Function

Studies on attached green macroalgae demonstrated that the bacterial communities were essentially assembled based on function (Burke et al., 2011a; Florez et al., 2017; Roth-Schulze et al., 2018). Blooming seaweeds usually have a higher metabolic level and are extremely sensitive to environmental stresses (Wang et al., 2012). To better understand the roles that the associated bacteria play in the blooming process, it is necessary to determine the relationships between bacterial communities and functions, environmental conditions and host status.

To address these issues, high-throughput sequencing of 16S ribosomal RNA (rRNA) genes was employed to analyze the community structures of bacteria associated with drifting *Sargassum* in the Yellow Sea. Moreover, a method for removal of epiphytic bacteria from the *Sargassum* surface was optimized to determine bacterial spatial distribution characteristics. In addition, the functions of bacterial communities were predicted to analyze their potential contributions to blooming.

## Materials and Methods

### Collection of Drifting *Sargassum* Samples

**FIGURE 1:**
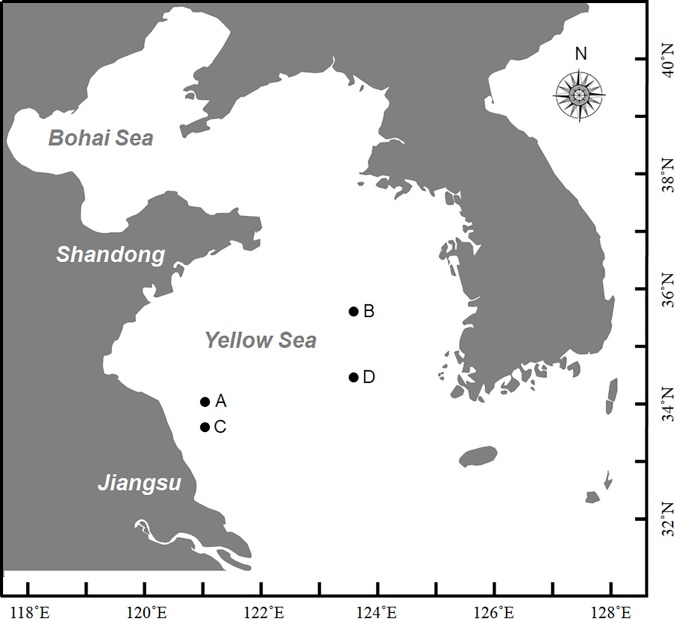
Sites for sampling in this study during Ke Xue III cruise. A and C represented nearshore sites with lower transparency where floating seaweeds were in brown color; B and D represented offshore sites with higher transparency where floating seaweeds were in yellow color.

**FIGURE 2:**
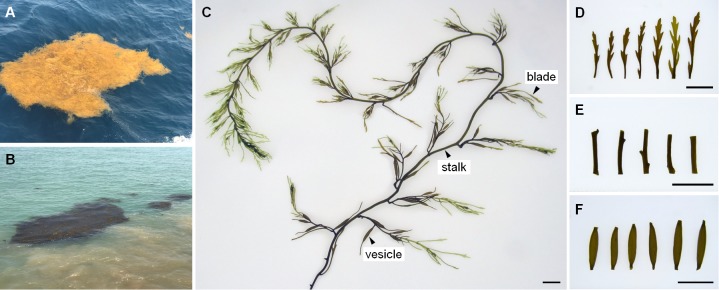
The status in field, morphology and tissues of drifting *Sargassum*. **(A)** In light yellow from offshore site. **(B)** In dark brown from nearshore site. **(C)** Thallus. **(D)** Blades. **(E)** Stalks. **(F)** Vesicles. Scale bars = 1 cm.

At the end of December in 2016, a large-scale of floating *Sargassum* was reported to drift southward from Shandong Peninsula to nearshore of Jiangsu (Xing et al., 2017). Then some biomass stayed in the nearshore area intertwined by *Pyropia* cultivation rafts, and others kept drifting and finally entered the offshore area. Until April when south wind occurred, these two parts of biomass all started to shift to drift northward (unpublished data). A cruise carried out by research vessel R/V Ke Xue III was conducted in early June to investigate the *Sargassum* bloom in the Yellow Sea. The floating *Sargassum* were sampled from both nearshore (A, C) and offshore (B, D) sites (Figure 1 and Table 1), in which the seawater transparency and seaweed color significantly varied. The dark brown seaweed was distributed nearshore on the surface of muddy seawater. Alternatively, the light yellow seaweed was always drifting in offshore seawater that was more transparent (Figure 2A,B). Approximately 400-ml surface water samples were also collected using CTD (SBE911, Sea-Bird Electronics, Inc., United States) from each site as environmental controls. The seawater transparency was measured using a secchi disk.

**Table 1:** Information on sampling sites surveyed in this study.

Site	Location	Date	Seawater transparency (m)	Seaweed color
A	34°00′N,121°00′E	June 08, 2017	0.1	Brown
B	35°30′N,123°30′E	June 22, 2017	14.0	Yellow
C	33°37′N,121°00′E	June 08, 2017	0.7	Brown
D	34°30′N,123°30′E	June 14, 2017	21.0	Yellow


### Molecular Identification and Chlorophyll Content Determination of Seaweed Samples

Five individuals from each sampling site were selected for molecular identification. Blades were washed with sterile seawater twice to remove any epiphytes and then homogenized by an electric tissue grinder (OSE-Y10, Tiangen, China). Total DNA was extracted with a Plant Genomic DNA Extraction Kit (Tiangen, China) and measured by spectrophotometer (Titertek Berthold, Germany). Nuclear-encoded ribosomal DNA internal transcribed spacer (ITS) 2 was amplified with a gradient thermal cycler (T960, Heal Force, China), following a previously described protocol (Yoshida et al., 2000). All PCR products were sub-cloned into pGEM-T cloning vector (Promega, United States) and sequenced by Sangon Biotech Co., Ltd. (China). Based on ITS2 sequence alignments from samples and representative taxa of brown seaweed using Clustal X 1.83, a phylogenetic neighbor-joining (NJ) tree was constructed by MEGA 5.0 using the Kimura 2-parameter model with 1000 bootstrap replications. To determine seaweed chlorophyll contents, 0.1 g of a segment from each individual was used for chlorophyll extraction and spectrophotometric analysis according to a previously described protocol (Jeffrey and Humphrey, 1975), and this was repeated in triplicate. After Levene test for the homogeneity samples variances via SPSS (v. 20), the difference of chlorophyll contents between brown and yellow samples were analyzed by One-way ANOVA using Microsoft Excel (2017).

### Optimization of Epiphytic Bacteria Removal From *Sargassum* Surface

A previous study on the green macroalgae *Ulva* showed that co-vortex of seaweed segments with silica sand was effective for removing and collecting most epiphytic bacteria; simultaneously, endophytic bacteria are prepared by grinding the remaining seaweed segments (Liu, 2015). Compared with *Ulva*, *Sargassum* has apparent tissue differentiation and a much tougher thallus surface. To test the effects on three kinds of *Sargassum* tissues (i.e., blade, vesicle, and stalk), the number of 15-min vortex replications were optimized from 1 to 10. The vortex suspension was composed of a piece of tissue (Figure 2C–F), silica sand with grain sizes of 125–250 μm, and sterilized seawater in a 1.5-ml centrifuge tube. After each round of vortexing for 15 min, the suspension was entirely renewed for the next round. From pipetted suspension, 10 μl was transferred to 2 ml of 2216E liquid media for culture at 28°C for 72 h; then, 200 μl of culture was transferred into a 96-well plate to measure the OD_600_ absorbance with a multi-functional microplate detector (M1000 Pro, Tecan, China). Because culturable bacteria account for a low percentage of environmental colonial samples (Amann et al., 1995), the removal effects were further detected by scanning electron microscopy (S-3400N, Hitachi, Japan) for tissues vortexed for 0, 5, or 10 replications. All tissues were fixed by 5% glutaraldehyde for 12 h, then gradient dehydration was carried out with 30, 50, 80, 90, and 100% ethanol at room temperature for 15 min, respectively. Observation was conducted at 5.00 kV and 1.50 K magnification. All treatments were conducted in triplicate.

### Isolation of Bacteria Associated With *Sargassum*

Isolation of both epiphytic and endophytic bacteria from *Sargassum* was conducted on the research vessel just after seaweed collection. Individuals of floating *Sargassum* were selected from nearshore and offshore sites, and washed with autoclaved seawater. Each of the three kinds of tissues (i.e., blades, vesicles, and stalks), were cut by autoclaved dissecting knives and then co-vortexed with sterilized silica sand five times, which was determined to be the optimal number of replications by the optimization procedure. For each sample, all vortex suspensions produced over the five rounds were combined and pre-filtrated by mixed cellulose ester filter membranes (Merck Millipore, United States) with 5.0-μm pores to remove silica sand and other contamination. Then, epiphytic bacteria were collected by a filter membrane with 0.22-μm pores. The surface-cleaned *Sargassum* tissue was homogenized by an electric tissue grinder (OSE-Y10, Tiangen, China), and sterilized seawater was added for resuspension. Using the same multi-grade filtration as for epiphytic bacteria, the potential endophytic bacteria and environmental bacteria were prepared for nearshore and offshore seawater samples. All filter membranes with 0.22-μm pores were kept in liquid nitrogen until return to the laboratory for subsequent molecular analysis.

### Microbial DNA Extraction and High-Throughput Sequencing

The microbial genomic DNA was isolated by a DNeasy PowerSoil Kit (QIAGEN, Netherlands) following the manufacturer’s instructions. To prevent potential amplification of 16S rRNA genes from organelle genomes of *Sargassum*, evaluations were conducted by aligning sequences of several pairs of universal primers with those of *S. horneri* mitochondrial (KJ938300.1) or chloroplast (KP881334.1) genomes. Finally, PCR amplifications of the V5–V7 region of the bacterial 16S rRNA gene were performed with primers 799F (5′-AACMGGATTAGATACCCKG-3′) and 1193R (5′-ACGTCATCCCCACCTTCC-3′) (Beckers et al., 2016). Sample-specific 7-bp barcodes were incorporated into the primers for multiplex sequencing. The PCR components contained 5 μl of Q5 reaction buffer (5×), 5 μl of Q5 High-Fidelity GC buffer (5×), 0.25 μl of Q5 High-Fidelity DNA Polymerase (5 U/μl), 2 μl (2.5 mM) of dNTPs, 1 μl (10 μM) of each forward and reverse primer, 2 μl of DNA template, and ddH_2_O added to 25 μl. Thermal cycling consisted of initial denaturation at 98°C for 2 min, followed by 25 cycles of denaturation at 98°C for 15 s, annealing at 55°C for 30 s, and extension at 72°C for 30 s, with a final extension at 72°C for 5 min. PCR amplicons were purified with Agencourt AMPure Beads (Beckman Coulter, United States) and quantified using the PicoGreen dsDNA Assay Kit (Invitrogen, United States). Amplicons were pooled in equal amounts, and paired-end (2 × 300 bp) sequencing was performed using the Illumina MiSeq platform with MiSeq Reagent Kit v3 at Shanghai Personal Biotechnology Co., Ltd. (China).

The Quantitative Insights into Microbial Ecology (QIIME, v1.8.0) pipeline was employed to process the sequencing data (Caporaso et al., 2010). The raw data of each sample were merged using Fast Length Adjustment of SHort reads (FLASH) to obtain raw tags (Magoc and Salzberg, 2011). After identifying and removing chimeric sequences, the effective tags were obtained and clustered to operational taxonomic units (OTUs) with a 97% similarity threshold. Taxonomic assignment of OTUs was performed by comparing sequences to the SILVA database.

### Bioinformatics and Statistical Analysis

Sequence data analyses were mainly performed using QIIME (v1.8.0) and R packages (v3.2.0). OTU-level alpha diversity analyses, including Chao1, Abundance-based Coverage Estimator (ACE), the Shannon index, and the Simpson index, were calculated using the OTU table in QIIME. The differences of the Chao1, ACE, Shannon index, and Simpson index between brown and yellow seaweed samples were calculated by One-way ANOVA after Bartlett test of homogeneity samples variances in R packages. OTU-level ranked abundance curves were generated to compare the richness and evenness of OTUs among samples. To investigate the structural variation of microbial communities across samples, beta diversity was estimated using UniFrac distance metrics (Lozupone and Knight, 2005; Lozupone et al., 2007) and visualized via Unweighted Pair Group Method with Arithmetic Means (UPGMA) hierarchical clustering using unweighted or weighted Unifrac Matrices (Ramette, 2007). Differences in Unifrac distances for pairwise comparisons among groups were determined using the Student’s *t*-test and the Monte Carlo permutation test with 1000 permutations via QIIME. Principal Component Analysis (PCA) using Euclidean distances was also conducted by R package “vegan” based on RDA function using the genus-level compositional profiles (Ramette, 2007). The significance of differentiation of microbiota structure among groups was assessed by Permutational Multivariate Analysis of Variance (PERMANOVA) (McArdle and Anderson, 2001) and Analysis of Similarities (ANOSIM) (Clarke, 1993; Warton et al., 2012) using the R package “vegan”. Taxa abundances at the phylum, class, order, family, genus, and species levels were statistically compared among samples or groups by Metastats (White et al., 2009) and visualized as column plots. The generalization error was estimated using 10-fold cross-validation. The expected “baseline” error was also included, which was obtained by a classifier that simply predicts the most common category label. Based on OTU data, microbial functions were predicted by Phylogenetic Investigation of Communities by Reconstruction of Unobserved States (PICRUSt) (Langille et al., 2013) and then categorized by Kyoto Encyclopedia of Genes and Genomes (KEGG) classification, and visualized in heatmap clustered by Hierarchical cluster via R package.

## Results

### Molecular Identification and Chlorophyll Content Determination of Seaweed Samples

**FIGURE 3:**
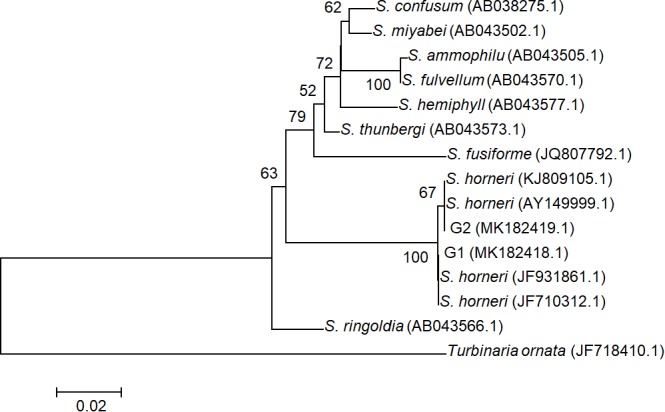
Phylogenetic NJ tree based on ITS2 alignment. Numbers at internal nodes indicate support values obtained using the bootstrap method with NJ. Scale indicates substitutions per site. G1 and G2 represented two genotypes of ITS2 sequences in *S. horneri*, representing intra-individual polymorphism in both yellow and brown samples.

All ITS2 sequences from 20 individuals were determined as two genotypes G1 (accession number MK182418.1) and G2 (accession number MK182419.1), which were distinguished by a single SNP and both deposited in GenBank. These two genotypes were found to represent intra-individual polymorphism in both yellow and brown samples. Phylogenetic analysis with G1, G2, and homologous sequences from other *Sargassum* species showed that all drifting seaweed belonged to a single species, *S. horneri* (Figure 3), which was consistent with the findings of a previous report (Su L. et al., 2018).

**FIGURE 4:**
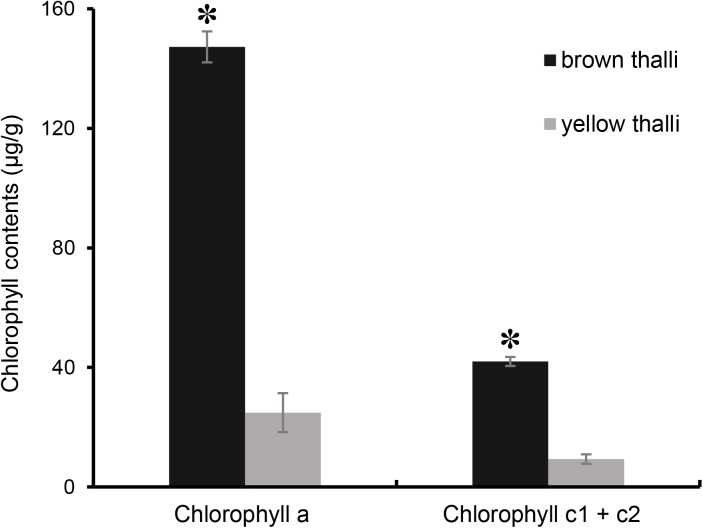
Comparison of chlorophyll contents between brown and yellow *S. horneri* thalli. The error bar represented the standard deviation, the asterisk above represented the significant differences.

However, the chlorophyll contents in brown and yellow *S. horneri* were significantly different (*P* = 1.41072E-05 for chlorophyll *a*, *P* < 0.01; *P* = 1.27422E-05 for chlorophyll c1 + c2, *P* < 0.01). As shown in Figure 4, the contents of chlorophyll *a* and c1 + c2 in brown *S. horneri* (147.30 μg/g ± 6.51 and 41.97 μg/g ± 1.559) were 5.93 and 4.5 times higher than those in yellow *S. horneri* (24.85 μg/g ± 5.195 and 9.33 μg/g ± 1.500), respectively, which indicates that the brown and yellow samples were in different physiological status.

### Optimization of Epiphytic Bacteria Removal From the *Sargassum* Surface

**FIGURE 5:**
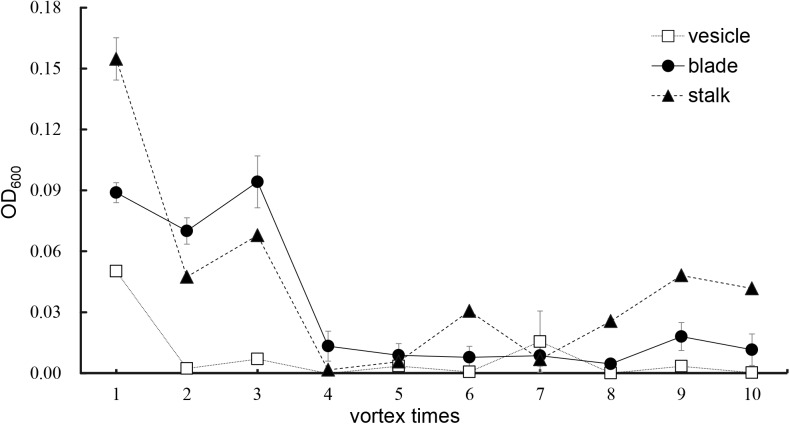
Culture method detection for removal effects of epiphytic bacteria from three kinds of *S. horneri* tissues.

**FIGURE 6:**
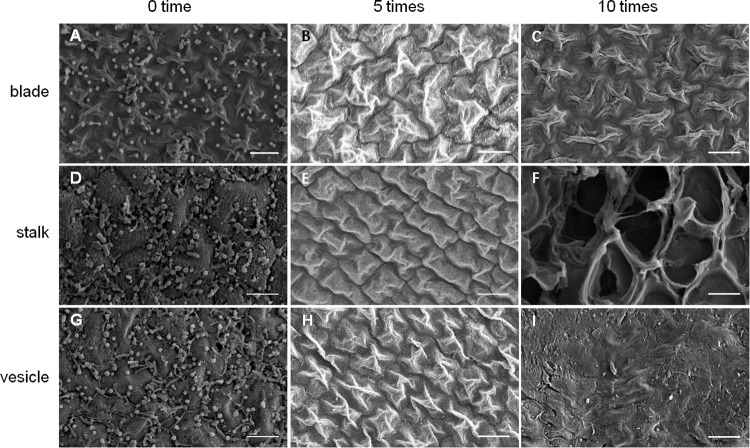
SEM detection for removal effects of epiphytic bacteria from three kinds of *S. horneri* tissues. **(A)** Blade blank control. **(B)** Blade vortexed for 5 times. **(C)** Blade vortexed for 10 times. **(D)** Stalk blank control. **(E)** Stalk vortexed for 5 times. **(F)** Stalk vortexed for 10 times. **(G)** Vesicle blank control. **(H)** Vesicle vortexed for 5 times. **(I)** Vesicle vortexed for 10 times. Scale bars = 5 μm.

As shown in Figure 5, an increase in number of co-vortex replications of seaweed tissues and silica sand was associated with a decrease in OD_600_ absorbance values in all kinds of tissues, which reflected the abundance of bacteria removed from each round of vortexing, and nearly reached to zero after five vortex replications, which indicates possible removal of all epiphytes. Subsequently, the abundance of removed bacteria slightly increased, especially in stalk groups, which indicates a potential release of endophytic bacteria from damaged *Sargassum* cells due to overtreatment. These results were consistent with those revealed by scanning electron microscopy, in which five rounds of vortexing could eliminate almost all epiphytic bacteria while retaining intact *Sargassum* cells, whereas 10 rounds of vortexing resulted in obvious damage to the seaweed surface, especially the stalk (Figure 6). Therefore, five replicates was determined to be optimal for subsequent vortex treatments.

### DNA Sequence Data and Profile of Microbiota

Annotation of sample names was based on the numbers and characters: 1, nearshore sites; 2, offshore sites; B, blade; S, stalk; V, vesicle; en, endophyte; ep, epiphyte; W, water sample. And this annotation was used throughout this paper including figure legends.

Based on Illumina MiSeq sequencing data, a total of 306,784 high-quality 16S rRNA gene sequences were obtained from 14 groups of samples, in which the percentage of sequences generated from organelle genomes of *S. horneri* were lower than 0.08%, which indicated that there were no notable interference to further analyses. All raw sequences were deposited in the NCBI Sequence Read Archive (SRA) under the accession number of PRJNA504763. According to the amplicon analyses, all sequences clustered into 19,867 OTUs at a 97% identity threshold, which were further classified into 525 bacterial groups at the genus level. Rarefaction curves and species accumulation curves demonstrated that the sequencing depth was sufficient to profile bacterial richness and diversity in all samples (Supplementary Figures S1, S2). The Chao1 and ACE estimators in Supplementary Table S1 indicated that the bacterial richness from the nearshore samples was much higher than that from offshore samples (Chao1: *P* = 0.013; ACE: *P* = 0.014). However, the Shannon and Simpson indexes exhibited similar bacterial diversity between these two types of sampling locations (Shannon: *P* = 0.144; Simpson: *P* = 0.140). It was shown that all samples were similarly dominated by sequences from Proteobacteria, Bacteroidetes, Actinobacteria, and Firmicutes at the phylum level (Supplementary Figure S3), but they were much more divergent at the genus level (Supplementary Figure S4).

### Bacterial Selectivity of the Seaweed Host Against Ambient Seawater

**FIGURE 7:**
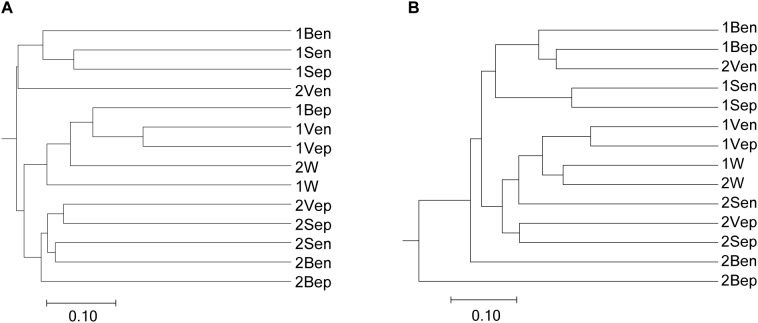
Dendogram of similarity of all samples clustered by the UPGMA method. **(A)** Unweighted. **(B)** Weighted. The scale bar shows the distance between clusters in UniFrac units.

**FIGURE 8:**
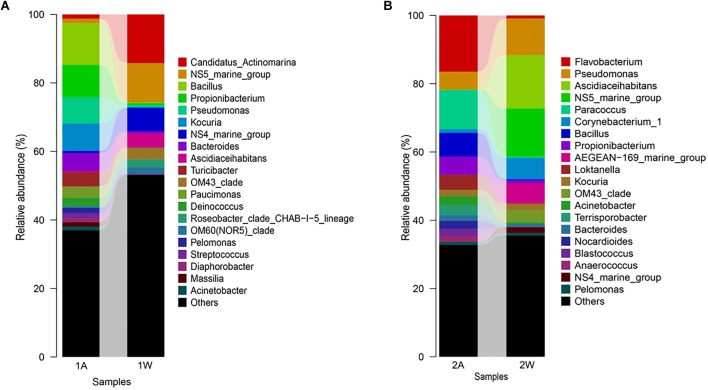
Bar-plot of bacterial composition of *S. horneri* and surrounding seawater at genus levels. **(A)** Nearshore. **(B)** Offshore. 1A: total bacteria isolated from brown *S. horneri*, 1W: total bacteria isolated from nearshore seawater, 2A: total bacteria isolated from yellow *S. horneri*, 2W: total bacteria isolated from offshore seawater.

High-throughput sequencing showed that the bacterial communities of drifting seaweed were clearly distinct from those of seawater regardless of if they were from nearshore or offshore habitats. As shown in Figure 7, the UPGMA trees constructed using weighted or unweighted Unifrac distance were similar, in which almost all samples of brown *S. horneri* were roughly separated from those of yellow *S. horneri*, whereas two ambient seawater samples, 1W and 2W, closely clustered together, which indicated obvious selectivity of seaweed host on their associated bacteria against the surrounding seawater. This was also shown clearly in the bar plot of Figure 8. In nearshore sites, the top five dominant bacterial genera from dark brown *S. horneri* were *Bacillus* (12.2%), *Propionibacterium* (9.48%), *Kocuria* (8.01%), *Pseudomonas* (7.65%), and *Bacteroides* (5.21%); however, they were present in extremely low amounts in seawater (0.299, 0.516, 0.179, 0.598, and 0.560%, respectively) (Figure 8A). A similar situation occurred in offshore sites, in which *Flavobacterium* (16.5%), *Paracoccus* (11.5%), *Bacillus* (6.97%), and *Propionibacterium* (5.27%) were mainly selected by light yellow *S. horneri*, whereas their percentages in seawater were 0.851, 0.483, 0.278, and 0.859%, respectively (Figure 8B). It was notable that, except for *Bacillus*, all dominant genera in seaweed were mainly composed of a single OTU.

**FIGURE 9:**
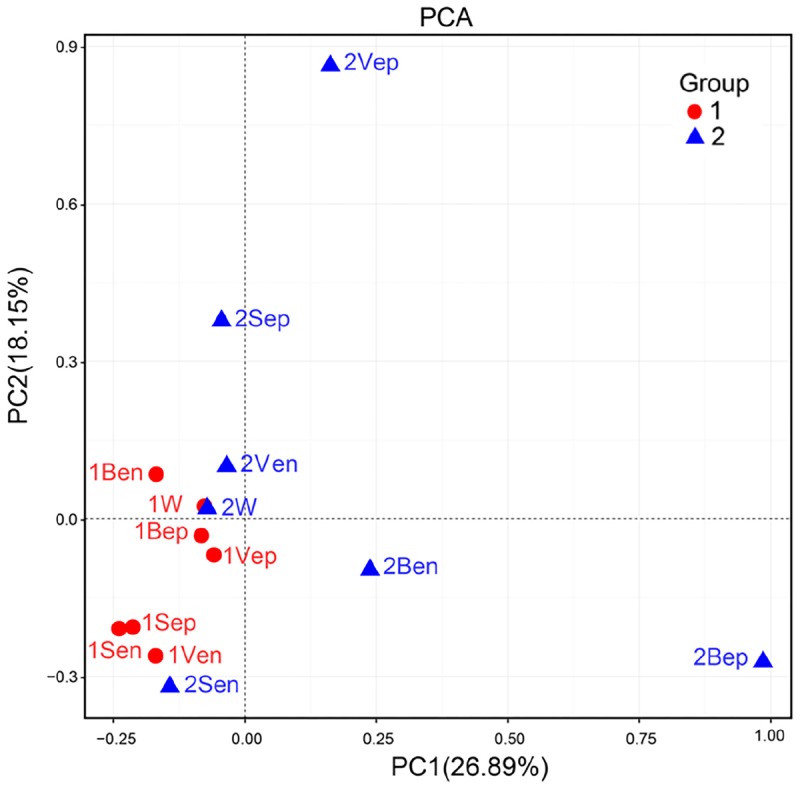
Principal Component Analysis (PCA) of the bacterial communities from all samples based on Euclidean distance at genus level.

Nevertheless, the selectivity of floating *S. horneri* on bacterial communities was not consistent between brown and yellow thalli. The Adonis/PERMANOVA results revealed that the bacteria associated with brown *S. horneri* were significantly different from those associated with yellow *S. horneri* (*P* = 0.006, unweighted UniFrac; *P* = 0.009, weighted UniFrac), which was visualized via PCA plot in which these two groups were distributed in different clusters (Figure 9). These two types of seaweed were sampled in different habitats; however, the two ambient seawater samples had similar bacterial structures (Figure 7, 9). Because brown and yellow *S. horneri* significantly varied in chlorophyll contents (Figure 4), we suggest that the seaweed selectivity on associated bacteria was highly affected by host physiological status.

### Spatial Distribution of Bacteria Associated With *S. horneri*

**FIGURE 10:**
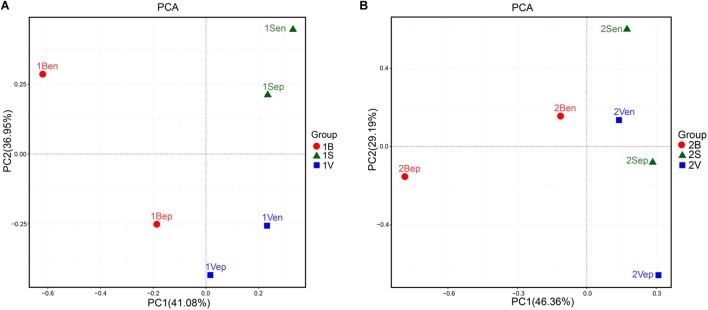
Principal Component Analysis (PCA) of the bacterial communities from blade, stalk, and vesicle of *S. horneri* based on Euclidean distance at genus level. **(A)** Brown *S. horneri*. **(B)** Yellow *S. horneri*.

As shown in Figure 10, the bacterial community structures of floating *S. horneri* seemed to be shaped by seaweed tissues, but in an algal physiological status-dependent manner. In brown *S. horneri*, samples from each of the three tissues (i.e., blade, vesicle, and stalk) clustered into a separate group (Figure 10A), and the Adonis/PERMANOVA results showed that the differences among the three tissues were close to significant (*P* = 0.067, unweighted UniFrac; *P* = 0.067, weighted UniFrac). By contrast, in yellow *S. horneri*, no distinct bacterial communities were detected among tissues (Figure 10B) and *P*-values were much higher (*P* = 0.600, unweighted UniFrac; *P* = 0.267, weighted UniFrac).

**FIGURE 11:**
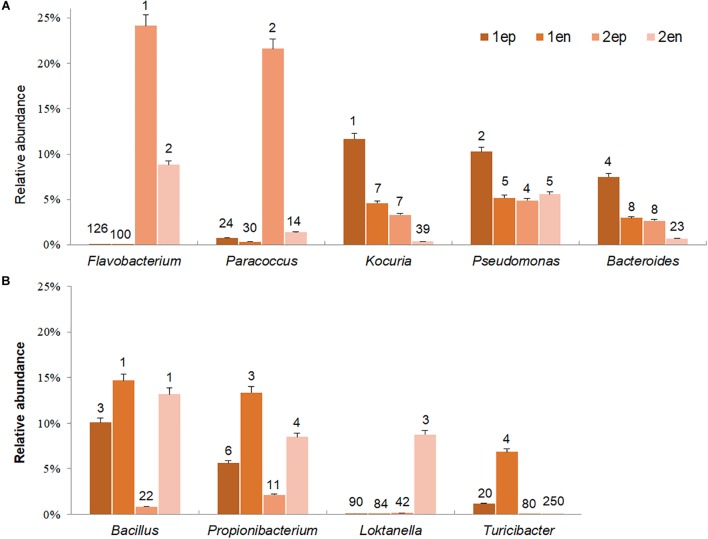
Potential dominant epiphytic and endophytic bacterial genera in brown and yellow *S. horneri*. **(A)** Epiphytic bacterial genera. **(B)** Endophytic bacterial genera. The number above column indicated the rank in abundance. 1ep: all epiphytic bacteria associated with brown thallus, 1en: all endophytic bacteria associated with brown thallus, 2ep: all epiphytic bacteria associated with yellow thallus, 2en: all endophytic bacteria associated with yellow thallus.

To further distinguish potential epiphytic and endophytic bacteria, the abundance and ranks of each dominant genus (abundance greater than 5% in any sample) were compared based on the data collected before and after grinding with the same types of seaweed samples. As shown in Figure 11, the abundances of *Flavobacterium* and *Paracoccus*, the two most dominant genera from yellow *S. horneri*, significantly decreased by 63.6 and 93.6% after grinding, respectively. Likewise, in brown *S. horneri*, the abundances of the three main dominant genera, *Kociria*, *Bacteroides*, and *Pseudomonas*, were reduced by 60.8, 60.2, and 49.5%, respectively (Figure 11A). Because violent friction effectively removed epiphytes, we suggest that bacteria from the above genera were most likely located on the surface of the seaweed thallus. Alternatively, some bacteria became dramatically much more predominant after grinding. Interestingly, no matter in yellow or brown *S. horneri*, *Bacillus*, and *Propionibacterium* significantly increased and became extremely dominant after removing epiphytes. In total, their abundances were increased by 60.6 and 64.8%, which indicates that they were the two main types of potential endophytes. Moreover, some genera were suspected to be specific endophytes of yellow or brown *S. horneri*. For example, *Turicibacter* was only detected in brown *S. horneri*, and their abundance increased by 82.8% after grinding; similarly, *Loktanella* abundance increased by 98.2% in yellow *S. horneri* (Figure 11B).

The endophytic bacteria dominated by *Bacillus* and *Propionibacterium* appeared to be much more consistent than epiphytes, regardless of host physiological status. *Bacillus* was mainly located inside vesicles (1Ven, 27.9%) or stalks (1Sen, 15.8%; 2Sen, 33.4%), whereas *Propionibacterium* occurred inside blades (1Ben, 36.9%) or vesicles (2Ven, 17.8%). In contrast, a sharp variation in composition of dominant epiphytes was observed between yellow and brown *S. horneri*. In yellow *S. horneri*, *Flavobacterium*, and *Paracoccus* occupied the surface of blades (2Bep, 63.0%) and vesicles (2Vep, 49.1%), respectively, with extremely high dominance. Alternatively, in brown *S. horneri*, *Kocuria* mainly occurred on the surface of vesicles (1Vep, 22.1%), and *Bacteroides* shared the blade surface (1Bep, 18.9%) with *Pseudomonas* (1Bep, 18.5%).

### Functional Metagenomic Predictions

**FIGURE 12:**
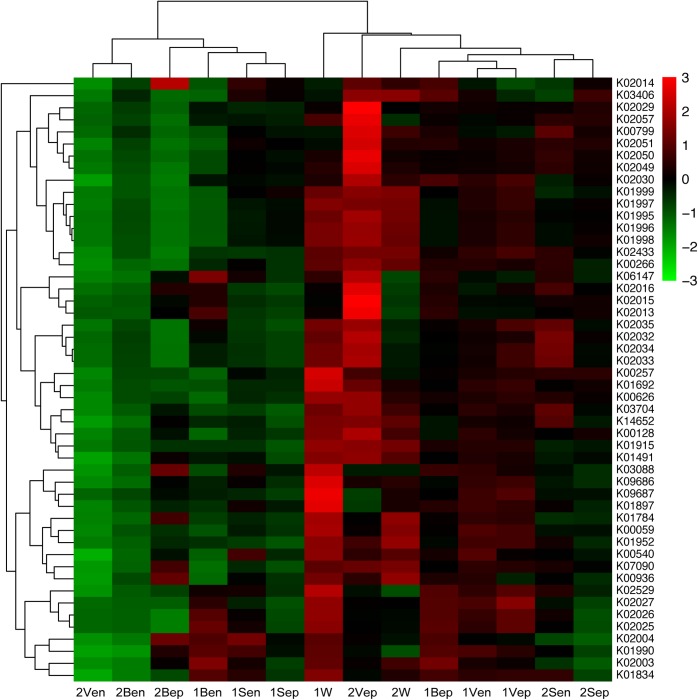
Heat-map of top 50 abundant functional proteins combined with the phylogenetic distribution of samples clustered by Hierarchical cluster. The color key scale represented the abundance of corresponding proteins. Each row represented the abundance distribution of right KEGG orthologous category (KO) among samples, and each column represented the abundance distribution of various KO in each sample whose name was shown at bottom. Lateral topology was contributed by the similarity of KO abundance distribution among different samples, and longitudinal topology was contributed by similarity of different KO species distribution in each sample.

After assessing the metabolic functions of bacterial communities by PICRUSt via the KEGG database, a total of 6,909 KEGG orthology (KO) categories were obtained and were assigned to 328 subsystems of 41 metabolic classes. As shown in Figure 12, the vertical topological structure illustrated the phylogeny of all samples based on the abundance of 50 most dominant functional proteins (Supplementary Table S2), which indicated that epiphytes were predicted to potentially have more functions than endophytes. Interestingly, the horizontal topological structure revealed that the nitrogen and iron metabolism-related proteins were enriched in both yellow and brown seaweed relative to seawater, which indicated that they might be crucial for golden tide blooms. These proteins were mainly classified into (1) nitrogen transport: peptide and nickel transporters (K02032–K02035), sulfate/nitrate/taurine transport system (K02049–K02051), and polar amino acid transport system (K02029 and K02030); and (2) iron transport: iron complex transport system (K06147 and K02013–K02016). Notably, these proteins were more dominant in yellow than brown seaweed. Alternatively, the sugar transport-related proteins, namely monosaccharide or oligosaccharide transporters (K02025–K02027), were only abundant in brown seaweed and nearshore seawater bacteria.

Analysis of spatial distribution of function proteins revealed that the nitrogen transport system was mainly dominant in 2Vep, 2Sen, 1Bep, and 1Vep; the iron transport system was mostly dominant in 2Vep, 2Bep, 1Ben, and 1Bep; and the sugar transport system was mainly distributed in 1Bep, 1Vep, 1Ben, 1Ven, and 1W. Overall, nitrogen and iron transport were predicted to be dominant functions in the epiphytic bacteria of vesicles and blades, especially in yellow *S. horneri*, and sugar transport mainly occurred on the surface of vesicles and blades in brown *S. horneri* and in nearshore seawater.

## Discussion

In this study, the community structures of bacteria associated with blooming *S. horneri* and ambient seawater were analyzed and compared. We found that the seaweed host exhibited obvious selectivity on associated bacteria, as the bacterial communities of drifting *S. horneri* were clearly distinct from those of seawater in both nearshore and offshore habitats (Figure 8). Algal–bacterial specificity has been widely reported in red seaweed (Longford et al., 2007; de Oliveira et al., 2012), green seaweed (Burke et al., 2011b; Hollants et al., 2013), brown seaweed (Lachnit et al., 2009; Michelou et al., 2013), and microalgae (Jasti et al., 2005); therefore, it is thought that the substance exchange between algae and associated bacteria may shape and maintain the algal–bacterial communities (Egan et al., 2013; Hollants et al., 2013).

However, seaweed selectivity on associated bacteria was not always stable and was affected by various environmental factors, such as season (Bengtsson et al., 2010; Tujula et al., 2010), salinity (Dittami et al., 2016), nutrients (Piggott et al., 2015), and even desiccation during low tide (Zhang et al., 2018). For blooming algae, such as *U. prolifera* (Liu et al., 2011), *Alexandrium tamarense* (Doucette, 1995), *Alexandrium catenella* (Zhou et al., 2018), and *Cochlodinium polykrikoides* (Park B.S. et al., 2015), the community structures of associated bacteria also varied at different bloom stages. In our two types of sampling locations with distinct physicochemical properties (Pan et al., 2018), the floating *S. horneri* obviously differed in color, chlorophyll contents (Figure 4), and bacterial communities. The seaweed chlorophylls contents have been proved to be related to their physiological status such as photosynthetic activity or growth rate (Zhang et al., 2013; Costa et al., 2017). Therefore, because the nearshore and offshore seawater microbiota were similar (Figure 7, 9), it was strongly indicated that host physiological status might shape the assembly of associated bacteria to meet the altered functional demands of the host (Lekunberri et al., 2012; Grottoli et al., 2018).

Studies on terrestrial plants showed that different parts of plants, like roots and leaves, may shape their specific bacterial compositions because of the substantial amount of variation in their respective local habitats (Sarria-Guzmán et al., 2016). For seaweed, although all parts of fronds live in the same seawater environment, the composition of associated bacteria among different parts or tissues also varied, which was also reported in *Chara vulgaris* (Ariosa et al., 2004), *Saccharina latissima* (Staufenberger et al., 2008), and *Ascophyllum nodosum* (Cundell et al., 1977). The lack of vascular connections was considered the key that might limit nutrient transport among tissues, thus leading to variant supplies of substances to associated bacteria (Goecke et al., 2010). In this study, such a tissue-specific distribution pattern of bacterial communities was also roughly shown in brown floating *S. horneri*, but was not apparent in yellow *S. horneri* (Figure 10). Further study with larger sample size is needed to confirm whether this pattern is affected by seaweed host physiological status.

The scanning electron microscopy and culture analysis results (Figure 5, 6) showed that co-vortex of *S. horneri* segments with silica sand might effectively remove most epiphytic bacteria. In particular, compared with other chemical methods involving strong oxidant (Kientz et al., 2011; Aires et al., 2012), this method provides a feasible approach for studying the community structure of endophytes with minimal interference (Liu, 2015). Moreover, we found that for each specific seaweed material, the protocol for parameter optimization was necessary, since overtreatment would result in the release of endophytes by damaging the seaweed cell walls. Using this new method, the potential dominant endophytic and epiphytic bacteria from golden tide-forming drifting *Sargassum* were suggested. As shown in Figure 11, most dominant endophytic bacteria of *S. horneri* belonged to *Bacillus* and *Propionibacterium*. Four dominate endophytic OTUs were identified in *Bacillus*, which shared high sequence identity with those endophytic *Bacillus* strains reported in the brown seaweed *Sargassum sabrepandum* (Ahmed et al., 2016), green seaweed *Ulva lactuca* (Nair et al., 2016), and red seaweed *Gracilaria dura* (Singh et al., 2011). Alternatively, only one endophytic strain was detected in *Propionibacterium*, and exhibited extremely high identity (99%) with *Propionibacterium acnes*, which lives inside higher plants (Tan et al., 2011; Sánchez-López et al., 2018). In addition, two other endophytic genera, *Loktanella* and *Turicibacter*, both of which only consisted of one OTU, were also found to be the main endo-symbionts in cnidaria (Doepke et al., 2012) and animal guts (Auchtung et al., 2016), respectively. Moreover, we found that epiphytic bacteria from *Flavobacterium*, *Paracoccus*, *Kocuria*, *Pseudomonas*, and *Bacteroides* mainly occupied the surface of *S. horneri*, and all of these genera are common among brown seaweed-associated bacteria (Staufenberger et al., 2008; Hollants et al., 2013; Park S.H. et al., 2015; Uzair et al., 2018). Interestingly, *Bacteroides* is known to be strictly anaerobic, we speculate that the presence of abundant polysaccharides at the surface of healthy seaweed thallus might likely protect *Bacteroides* from exposure to oxygen.

It is worth noting that, following a change in environment and host status, the composition of dominant endophytes was quite consistent, whereas that of major epiphytes substantially changed; this could be main reason for the significant variation between the bacterial communities of yellow and brown *S. horneri*. Several studies reported that the composition of algal endophytes was very stable and unique (Hollants et al., 2011a), and has been used to reveal the invasion route of algal hosts (Aires et al., 2013). The identification of endophytes in drifting *S. horneri* may inspire additional approach, because the origin of golden tides in the Yellow Sea is still unknown (Komatsu et al., 2014b; Qi et al., 2017).

Metabolic protein prediction (Figure 12) revealed that the bacteria associated with seaweed differed from those in seawater in both phylogeny and function. Moreover, their functions in nitrogen and iron transport were dominant in seaweed regardless of host status, which indicated that they might play crucial roles both in golden tide blooms and in the survivals of seaweed under stress. Because the bacterial communities in yellow and brown *S. horneri* were significantly different in phylogeny, we suggest that algae-associated bacteria tended to be assembled by function, which was consistent with the competitive lottery theory (Burke et al., 2011b). Interestingly, the abundances of nitrogen and iron transport proteins were much higher in yellow *S. horneri*, whereas sugar transport proteins were dominant in brown *S. horneri*; this demonstrated an obvious correlation among environmental characteristics, host demands, and bacterial community functions.

Nitrate accounted for 80–100% of dissolved inorganic nitrogen (DIN) in the South Yellow Sea (Gao et al., 2012). According to the data collected from the same cruise, the nitrate concentration was approximately 30 μM in the nearshore locations but close to zero in the offshore locations (Pan et al., 2018), which indicated that yellow *S. horneri* was under nitrogen deficiency stress compared with brown *S. horneri*. We found that the nitrogen transport functions were extremely dominant on the surface of vesicles in yellow *S. horneri*, and this appeared to be associated with a single OTU from *Paracoccus*, which was extremely dominant (49%) among epiphytes. Active nitrate transport was reported in *Paracoccus denitrificans*, a closely related strain capable of nitrate assimilation (Goddard et al., 2017), and several closely related strains of *Paracoccus* had urease activity (Liu et al., 2008; Shen et al., 2012), which could be influenced by the peptide and nickel transporters (Hiron et al., 2010). Therefore, we speculated that nitrogen transport enhancement by the epiphytes might improve host growth nearshore, and help protect the host from nitrogen deficiency-based stress offshore by enriching some unique OTUs with enhanced functions. However, the potential mechanism of nitrogen transmission between seaweed and bacteria was still unclear.

As predicted, the iron complex (siderophore iron) outer membrane receptor protein (OMP) and iron complex transport system proteins were abundantly distributed in epiphytic bacteria of blades and vesicles, respectively, especially in yellow *S. horneri*. Iron is essential for chlorophyll synthesis in seaweed. According to the data collected in the Yellow Sea in spring 2015, the concentration of dissolved iron was approximately 70 nM nearshore, but as low as approximately 12 nM in offshore seawater (Su et al., 2015; Su H. et al., 2018). Studies showed that *S. horneri* was yellow in 14.4 nM of dissolved iron, and turned brown above 43 nM, which indicates increased chlorophyll content (Miki et al., 2016). Thus, we speculate that the yellow *S. horneri* were also under iron deficiency stress compared with brown *S. horneri*. Approaches on iron concentration mechanism in brown seaweed showed that, the carboxylate group of alginate on the thallus surface could absorb iron or possibly exchange iron with bacterial siderophores (Miller et al., 2016). We found that *Flavobacterium* highly dominated the surface of blades and *Paracoccus* dominated the surface of vesicles, and both were prominent in iron transport. TonB-dependent OMP, which was crucial for transport of the siderophore–iron complex, has been recognized in several closely related strains of *Flavobacterium* (Møller et al., 2005; Guan et al., 2013) and several closely related *Paracoccus* strains had strong abilities to produce siderophores (Bergeron et al., 1985; Bergeron and Weimar, 1990; Sedlácek et al., 2009). These two dominant OTUs might play key roles in supplying iron to hosts under stress. However, further study is needed to determine whether siderophore-mediated iron transmission occurs between seaweed and bacteria.

In addition, sugar transport was predicted to be abundant on both the surface of brown *S. horneri* and in nearshore seawater, which has been proved in other seaweed (Burke et al., 2011b; Zhang and Wang, 2017). A study indicated that the seaweed surface continued to secrete polysaccharides into surrounding seawater, which provides a carbon source for bacteria (Goecke et al., 2010). Consistent with the function prediction, we found that the surface of brown *S. horneri* was much more slippery than that of yellow *S. horneri*, which might arise from a low metabolic level and deficient polysaccharide secretion in yellow *S. horneri*.

It should be emphasized that the metabolic function analysis was only based on bioinformatics prediction by PICRUSt, the accuracy of PICRUSt relies on the availability of completely sequenced genomes for the representative organisms (Wilkinson et al., 2018), and this method might lead to low accuracy for function prediction of endophytes (Langille et al., 2013). In this study, *Bacillus* and *Propionibacterium* OTUs were identified as dominant endophytic bacteria in drifting *S. horneri*, but no strong functions were predicted. However, *Bacillus* has been widely reported to be plant endophytes with various abilities that enhance the host’s competitive advantage, including promotion of host growth (Li et al., 2018), bioleaching of iron (Sun et al., 2017), producing IAA and fixing nitrogen to promote sprouting for seaweed (Singh et al., 2011), and releasing antibacterial substances to protect hosts against disease (Pathak et al., 2012). Therefore, endophytes should be investigated further, because several *Bacillus* strains in this study have been successfully isolated and cultured. In addition, nitrogen and iron metabolism still need to be verified in the field, by RT-PCR of target genes, and metagenomics analysis, because of the high proportion of unknown OTUs observed in bacterial communities.

## Conclusion

In this study, the community structure and functions of bacteria associated with drifting *S. horneri* were analyzed. We showed that the seaweed host had obvious selectivity on associated bacteria, which were assembled via functions rather than phylogeny. The proteins involved in nitrogen and iron transporters dominated the predicted functions, which indicated that the microbial community may greatly contribute to algal host blooms. In response to weakness of host status, the epiphytic OTUs extensively varied, whereas the dominant functions were consistent but at elevated intensities, which might protect the host from nitrogen or iron deficiency-based stress. Alternatively, the composition of endophytic bacteria remained consistent, and they potentially play key roles in rapid growth of the host. We propose that host physiological status, with different intensities of functional demands related to variable environmental conditions, may be a critical factor that influences epiphytic bacterial community assembly. This study provided new insights into the structure and potential functions of associated bacteria with golden tide blooms.

## Author Contributions

PJ and XM conceived and designed the experiments. XM and CW performed the experiments. JZ, XM, and PJ analyzed the data. CW and TY collected the samples. XM and PJ wrote the original draft. PJ revised the manuscript. All authors reviewed and approved the final manuscript.

## Conflict of Interest Statement

The authors declare that the research was conducted in the absence of any commercial or financial relationships that could be construed as a potential conflict of interest.
