# Supplementary material for: Community Structure of Bacteria Associated With Drifting Sargassum horneri, the Causative Species of Golden Tide in the Yellow Sea
Source: Front Microbiol. 2019 May 28;10:1192. doi: 10.3389/fmicb.2019.01192 (PMC6546727; doi:10.3389/fmicb.2019.01192)
Supplement: Supplementary file 1 [file Data_Sheet_1.PDF]

Supplementary Material

Supplementary Tables

Supplementary Table 1. Chao1 and ACE richness estimator, Shannon and Simpson diversity index of samples. Annotation for the numbers and characters in sample names was used throughout this paper. 1: nearshore sites, 2: offshore sites, B: blade, S: stalk, V: vesicle, en: endophyte, ep: epiphyte. W: water sample.

Samples	Chao1	ACE	Shannon	Simpson
1Ben	301.31	306.21	4.68	0.874564
1Bep	544.12	557.38	5.90	0.962499
1Sen	438.00	455.38	6.01	0.966742
1Sep	444.02	445.08	6.70	0.978963
1Ven	699.56	761.33	6.05	0.959929
1Vep	583.47	626.00	5.49	0.931418
1W	561.71	574.61	5.56	0.944694
2Ben	304.26	316.83	5.46	0.946968
2Bep	269.08	277.41	3.72	0.772272
2Sen	319.02	331.25	4.88	0.917699
2Sep	340.66	365.70	4.50	0.909784
2Ven	532.00	532.00	7.33	0.983515
2Vep	231.50	245.33	3.54	0.756598
2W	334.10	345.82	5.08	0.934883

Supplementary Table 2. The predicted top 50 dominated proteins in all samples.

KEGG orthology	KEGG annotation
K02051	sulfonate / nitrate / taurine transport system substrate-binding protein
K02050	sulfonate / nitrate / taurine transport system permease protein
K02049	sulfonate / nitrate / taurine transport system ATP-binding protein
K02057	simple sugar transport system permease protein
K03088	RNA polymerase sigma-70 factor, ECF subfamily
K02030	polar amino acid transport system substrate-binding protein
K02029	polar amino acid transport system permease protein
K01834	phosphoglycerate mutase [EC:5.4.2.1]
K02035	peptide / nickel transport system substrate-binding protein
K02034	peptide / nickel transport system permease protein
K02033	peptide / nickel transport system permease protein
K02032	peptide / nickel transport system ATP-binding protein
K07090	None
K02004	None
K02003	None
K00936	None
K00540	None
K00257	None

K02027	multiple sugar transport system substrate-binding protein
K02026	multiple sugar transport system permease protein
K02025	multiple sugar transport system permease protein
K01491	methylenetetrahydrofolate dehydrogenase (NADP+) / methenyltetrahydrofolate cyclohydrolase [EC:1.5.1.5 3.5.4.9]
K03406	methyl-accepting chemotaxis protein
K01897	long-chain acyl-CoA synthetase [EC:6.2.1.3]
K02529	LacI family transcriptional regulator
K02016	iron complex transport system substrate-binding protein
K02015	iron complex transport system permease protein
K02013	iron complex transport system ATP-binding protein [EC:3.6.3.34]
K02014	iron complex outermembrane recepter protein
K01524	guanosine-5'-triphosphate,3'-diphosphate pyrophosphatase [EC:3.6.1.40];exopolyphosphatase / guanosine-5'-triphosphate,3'-diphosphate pyrophosphatase [EC:3.6.1.11 3.6.1.40]
K00799	glutathione S-transferase [EC:2.5.1.18]
K01915	glutamine synthetase [EC:6.3.1.2]
K00266	glutamate synthase (NADPH/NADH) small chain [EC:1.4.1.13 1.4.1.14]
K01692	enoyl-CoA hydratase [EC:4.2.1.17]
K03704	cold shock protein (beta-ribbon, CspA family)
K03496	chromosome partitioning protein
K01999	branched-chain amino acid transport system substrate-binding protein

K01998	branched-chain amino acid transport system permease protein
K01997	branched-chain amino acid transport system permease protein
K01996	branched-chain amino acid transport system ATP-binding protein
K01995	branched-chain amino acid transport system ATP-binding protein
K06147	ATP-binding cassette, subfamily B, bacterial
K02433	aspartyl-tRNA(Asn) / glutamyl-tRNA (Gln) amidotransferase subunit A [EC:6.3.5.6 6.3.5.7]
K09687	antibiotic transport system ATP-binding protein
K00128	aldehyde dehydrogenase (NAD+) [EC:1.2.1.3]
K00626	acetyl-CoA C-acetyltransferase [EC:2.3.1.9]
K01990	ABC-2 type transport system ATP-binding protein
K00059	3-oxoacyl-[acyl-carrier protein] reductase [EC:1.1.1.100]
K14652	3,4-dihydroxy 2-butanone 4-phosphate synthase / GTP cyclohydrolase II [EC:4.1.99.12 3.5.4.25]

Supplementary Figures

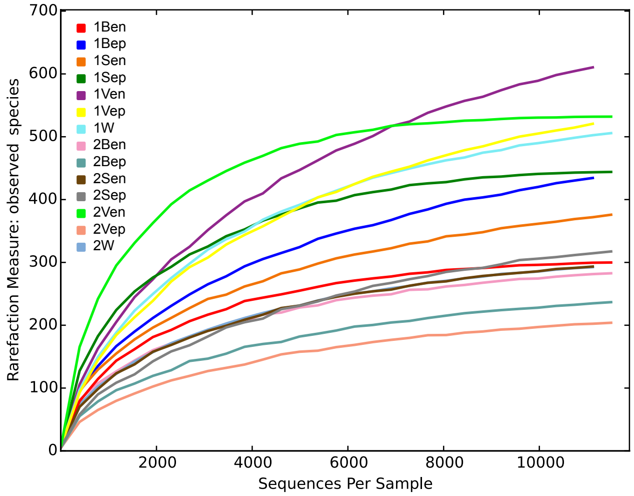

Supplementary Figure 1. Rarefaction curves for the number of OTUs from 14 groups of samples.

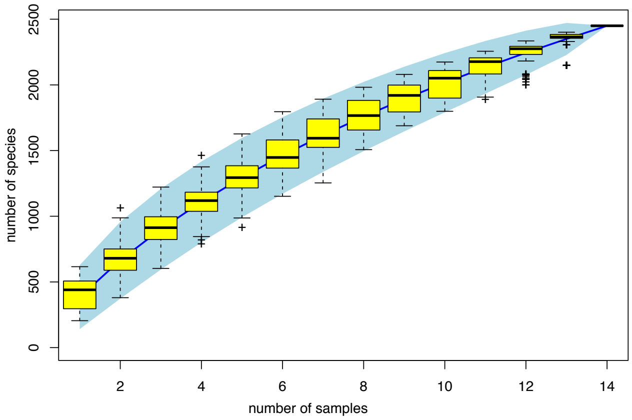

Supplementary Figure 2. Species accumulation curves for the number of OTUs from 14 groups of samples.

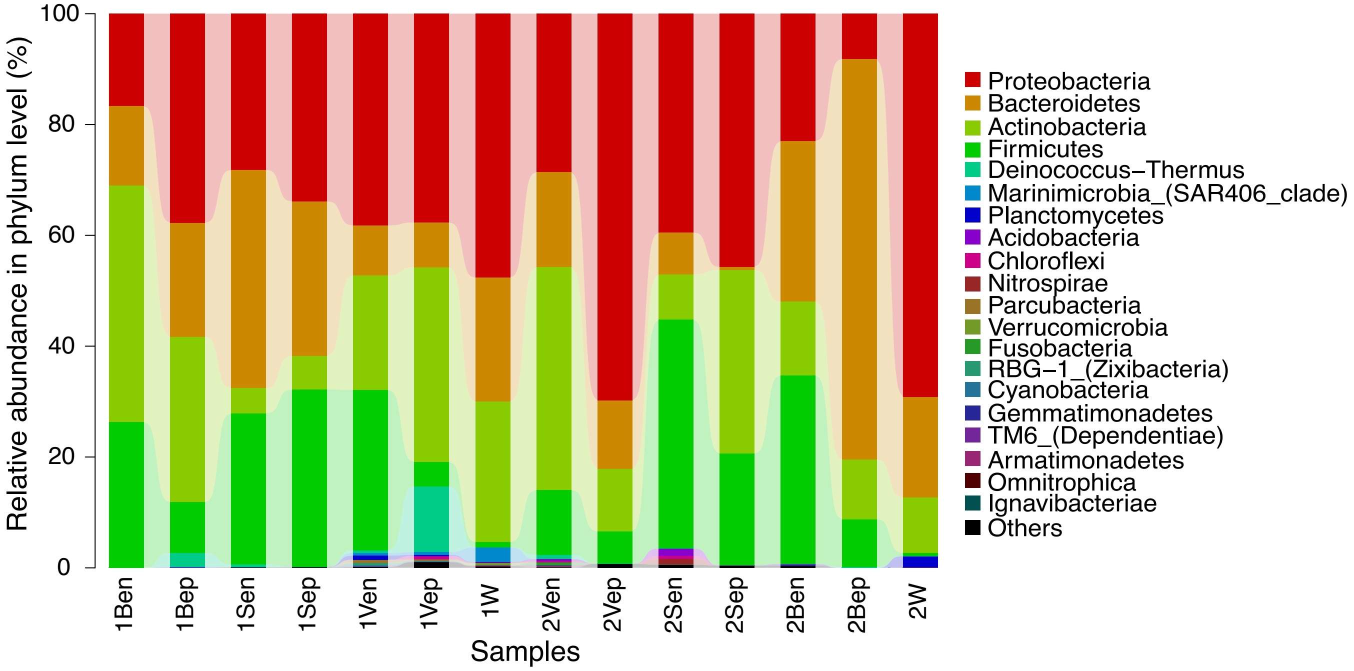

Supplementary Figure 3. The bacterial community structure of 14 groups of samples at phylum levels.

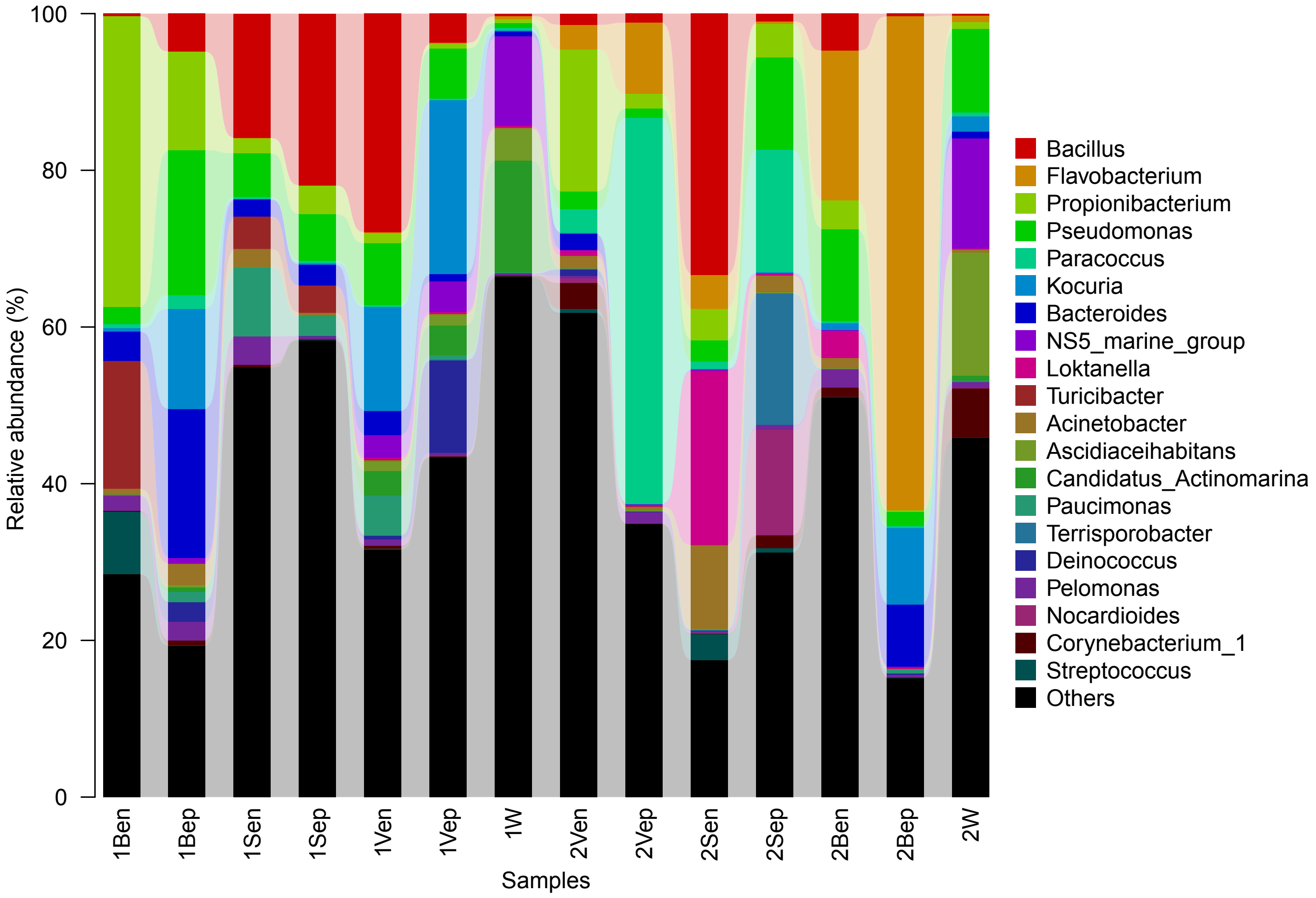

Supplementary Figure 4. The bacterial community structure of 14 groups of samples at genus levels.
